# Too Much Materia Medica, Etc.

**Published:** 1880-10-20

**Authors:** A. G. Smythe

**Affiliations:** Miss.


### TOO MUCH MATERIA MEDICA—TOO MANY PREPARATIONS OF CINCHONA BARK—THE CULTURE OF THE CINCHONA TREE IN THE UNITED STATES.

BY A. G. SMYTHE, M.D., OF MISS.

There is a sensible question in the Western Lancet, when calling attention to the new preparation quinamine. "When will this diabolism cease, and will the traffickers in remedyism ever let up their schemes of crowding into the race their tiresome innovations, and endeavoring to compel physicians to prescribe their silly and useless nostrums?" It is not in the spirit of opposition to the proper and legitimate advance in the march of discovery, or to check the enterprise of the chemist and pharmacist, all of which are right in their proper way and place, but there is a point beyond which endurance ceases to be a Christian virtue. The different preparations of cinchona bark which are and have been presented with all the contortions and distortions in the vocabulary and nomenclature of remedial agents in therapeutics, are not simply an annoyance, but have become disgusting to the sober philosophic physician, who has not the time at his command to become familiar with the different names and the peculiar definitions of the different preparations of cinchona bark, which are now offered to the general practitioner, to say nothing of an opportunity to make a sufficient trial to ascertain whether they can be substituted for the more familiar preparations, whose character is already established. And was he to attempt to make the trial in private practice, it would cost him his professional character, if it was known that he was making an experiment of the kind, unless when it was impossible to procure the usual standard articles.

While the people at large (especially in the rural or country districts) are willing that their physicians be equally well skilled in all that pertains to their profession, they, at the same time, have a great aversion to the making of experiments upon themselves.

There is at present in the South and West a violent prejudice against the use of the sulphate cinchonidia, and in many localities it is only used covertly. The only way to get out of this dilemma will be to fall back on the crude powdered cinchona bark. Nothing can be cheaper that is prepared from it. Whilst your correspondent has at all times been an advocate for improvement in all things, and holds to the doctrine of progression now, he is forced to the conclusion that the introduction of such a host of new remedies and new preparations, while all or most of the old are retained, is swelling the catalogue of articles in the materia medica to an alarming extent, and unless there is some check upon it, where or when will it stop? It must have an end, or all the knowledge of the physician will be swallowed up in it. And yet we must keep up with all the departments of the science of medicine, and the collateral sciences, possess general intelligence and culture, as well as be gentlemen of leisure.

Why, the articles of the materia medica have accumulated into a mass of such colossal proportions, that to thoroughly comprehend and understand the natural, commercial, chemical and therapeutical histories of the same, would consume the greater part of the manhood life of most of men, afford almost no time for the acquirement of the necessary knowledge in the other important and indispensable departments of the science of medicine. Unless there is some check put upon this thing, everything in each of the great kingdoms of nature, mineral, vegetable, animal and aerial, will be incorporated in the catalogue of materia medica, a thing of such magnitude that no human understanding could comprehend it; the creator only could know it. In short, the only way to correct the abuse is to select the fittest, select a sufficient number of well tried standard articles in the different departments of materia medica, and if not supposed to be advisable to discard all, or even a part of the secondary list; do not require candidates for the doctorate to be familiar with the use of a mass of useless remedies, when their time could be spent with so much more profit in the other departments of medicine.

I set out to say something about the growing evil, for evil it is, of swelling the materia medica to such an extent; have said more than was my intention, but could not say less and say anything, and as I commenced upon cinchona, will close upon it.

There is at this time a great desire in the medical world to procure a substitute for the present high-priced preparations of cinchona bark. Many attempts have been made to introduce articles of various kinds, all of which have in a measure failed. It is now a well established fact, that with our present knowledge, no article in the materia medica can fill the place of cinchona and its preparations. To meet the wish and necessity—for necessity it is—chemists and pharmacists have taxed their skill and ingenuity, if not to their utmost, to a degree that has become annoying to the general practitioner. What your contributor desires to call attention to at this time is the introduction and culture of the cinchona tree in the United States. That it can be successfully cultivated in the elevated regions of S. W. Virginia, Western North Carolina, North Georgia and Alabama, also the mountainous portions of the Pacific States, is borne out by the various reports of its successful and profitable culture in the British East Indies. Being so truly convinced of the importance of this subject, your contributor presented the following resolution, which was unanimously adopted, at the late meeting of the Mississippi State Medical Association, at Vicksburg, Miss., in April, 1880.—Vide Transactions of the Association, 1880, page 30.

"*Resolved*, That the Mississippi State Medical Association respectfully request the Senators and Representatives from the State of Mississippi, in the Congress of the United States, to present a bill to create a Scientific Commission to consider the practicability of the introduction of the cinchona tree in the mountainous districts of the United States; the enterprise to be under the patronage of the Agricultural (or other) department of the Government."

Your contributor is of opinion that if a small portion of the capital, brain and labor now devoted to the manufacture and introduction of the many preparations of cinchona was devoted to the support of the foregoing resolution, that it would, in all probability, advance all the interests, wishes and intentions of all classes of American citizens, as well as the interests of humanity, in the manufacture of the manifold preparations of cinchona; would open a new and profitable industry, also bring into use and occupation large tracts and bodies of unsettled and unoccupied lands in the wild and mountainous portions of the country, to say nothing of the benefit of an independent source of supply of this now indispensable article of the materia medica. Much more might be said in this connection upon this subject, but I forbear for the present, reserving the right to add whatever may be necessary in the future, in advocating the proposition embodied in the resolution offered by your contributor trusting that what has already been said upon the subject will invite a discussion from sources that are better informed and more capable of setting forth the merits of the proposition than your humble but devoted contributor.

It is to be hoped that the apparent objection to the great number of preparations of cinchona will not be construed so as to ground the opinion that there is or was any disposition to oppose the legitimate advance in the march of inquiry, discovery or improvement. Such is not, nor was not, the intention in the most remote sense, but to call attention to what is a growing evil and abuse in certain quarters. It is only by philosophic inquiry and experiment that discoveries and improvements are made, except when by accident. The time has come when it is necessary that there should be a check upon this abuse.

The foregoing explanation was necessary to prevent a false construction upon the language used at the outset of this article.

